# Karyotype Differentiation in Cultivated Chickpea Revealed by Oligopainting Fluorescence *in situ* Hybridization

**DOI:** 10.3389/fpls.2021.791303

**Published:** 2022-01-25

**Authors:** Alžběta Doležalová, Lucia Sládeková, Denisa Šimoníková, Kateřina Holušová, Miroslava Karafiátová, Rajeev K. Varshney, Jaroslav Doležel, Eva Hřibová

**Affiliations:** ^1^Centre of the Region Hana for Biotechnological and Agricultural Research, Institute of Experimental Botany of the Czech Academy of Sciences, Olomouc, Czechia; ^2^Department of Cell Biology and Genetics, Faculty of Science, Palacký University, Olomouc, Czechia; ^3^Centre of Excellence in Genomics and Systems Biology, International Crops Research Institute for the Semi-Arid Tropics (ICRISAT), Hyderabad, India; ^4^State Agricultural Biotechnology Centre, Centre for Crop and Food Innovation, Murdoch University, Murdoch, WA, Australia

**Keywords:** *Cicer arietinum* L., kabuli type, desi type, oligopainting FISH, chromosome identification, chromosome translocation

## Abstract

Chickpea (*Cicer arietinum* L.) is one of the main sources of plant proteins in the Indian subcontinent and West Asia, where two different morphotypes, desi and kabuli, are grown. Despite the progress in genome mapping and sequencing, the knowledge of the chickpea genome at the chromosomal level, including the long-range molecular chromosome organization, is limited. Earlier cytogenetic studies in chickpea suffered from a limited number of cytogenetic landmarks and did not permit to identify individual chromosomes in the metaphase spreads or to anchor pseudomolecules to chromosomes *in situ*. In this study, we developed a system for fast molecular karyotyping for both morphotypes of cultivated chickpea. We demonstrate that even draft genome sequences are adequate to develop oligo-fluorescence *in situ* hybridization (FISH) barcodes for the identification of chromosomes and comparative analysis among closely related chickpea genotypes. Our results show the potential of oligo-FISH barcoding for the identification of structural changes in chromosomes, which accompanied genome diversification among chickpea cultivars. Moreover, oligo-FISH barcoding in chickpea pointed out some problematic, most probably wrongly assembled regions of the pseudomolecules of both kabuli and desi reference genomes. Thus, oligo-FISH appears as a powerful tool not only for comparative karyotyping but also for the validation of genome assemblies.

## Introduction

Chickpea (*Cicer arietinum* L.) is an important legume crop and one of the main sources of dietary proteins in South Asia and sub-Saharan Africa, where two different morphotypes, desi and kabuli, are grown. Chickpea originated in southeastern Turkey and was domesticated in the Middle East about 10,000 years ago, approximately during the same time as wheat, barley, pea, and lentil (Redden and Berger, 2007). The domestication bottleneck and human selection resulted in a narrow genetic basis of cultivated chickpeas, which hampers breeding climate-resilient high-yielding varieties (Abbo et al., 2003; Varshney et al., 2019). The low level of genetic diversity in chickpea was demonstrated using a variety of molecular methods (Thudi et al., 2011; Hiremath et al., 2012; Roorkiwal et al., 2014; Gupta et al., 2017). In contrast, a large genetic diversity in wild *Cicer* species was revealed using classical molecular markers such as RAPD, ISSR, and SSR (Sudupak et al., 2002; Rajesh et al., 2003; Upadhyaya et al., 2008).

Draft genome assemblies of both morphotypes were produced recently (Varshney et al., 2013; Ruperao et al., 2014; Parween et al., 2015), establishing a solid foundation for the development of molecular tools to speed-up chickpea breeding. Draft genome sequence of desi type was created from a combination of 454/Roche and Illumina sequence reads (Jain et al., 2013), improved by mate-pair Illumina sequence data (Parween et al., 2015) and scaffolded using bacterial artificial chromosome (BAC)-end sequences. Scaffolds were ordered into pseudomolecules by use of marker sequences from interspecific genetic linkage maps (Gaur et al., 2015). The desi assembly represented 519 Mb, corresponding to 59.8% of its genome (considering 1C ∼ 868 Mb, Ruperao et al., 2014). Draft genome sequence of kabuli type of chickpea was constructed from Illumina sequence data and BAC-end sequences. The pseudomolecules were assembled using DNA markers (Thudi et al., 2011; Hiremath et al., 2012) that differed from those used in the desi genome sequence project. Finally, the scaffolds were ordered based on the synteny with the *Medicago truncatula* genome (Varshney et al., 2013). The draft sequence of kabuli captured 532 Mb, 60.3% of the estimated genome size (Ruperao et al., 2014). The use of a different strategy to order the scaffolds into pseudomolecules resulted in their different orientation between desi and kabuli types (Parween et al., 2015). Moreover, neither desi nor kabuli pseudomolecules were anchored to chromosomes.

Subsequent re-sequencing of multiple accessions led to the identification of a large number of DNA markers, some of which are associated with important traits, and the identification of candidate genes related to important agronomic characters (Thudi et al., 2016; Varshney et al., 2019). These efforts were accompanied by the development of high-density genetic maps (Roorkiwal et al., 2018; Deokar et al., 2019; Barmukh et al., 2021) to complete the knowledge of the chickpea genome organization and to deliver resources needed for the application of marker-assisted selection in chickpea improvement.

The progress in genome mapping and sequencing contrasts with limited knowledge of the chickpea genome at the chromosomal level, including the long-range molecular chromosome organization. This knowledge gap prevents the anchoring of individual pseudomolecules of genome assembly to particular chromosomes, precludes comparative karyotype analysis between the chickpea morphotypes and related species, impedes the analysis of the behavior of individual chromosomes during meiosis, and the identification of chromosome structural changes. The relatively small nuclear genome of chickpea (∼748–882 Mb) (Arumuganathan and Earle, 1991; Ruperao et al., 2014) is divided into eight submetacentric or metacentric chromosomes (Ohri and Pal, 1991). Out of the eight chromosomes, only two largest chromosomes, one bearing nucleolus organizer region, and the smallest chromosome could be identified based on morphology and presence of heterochromatin (Karafiátová et al., 2017). Large heterochromatin blocks comprise two main chickpea satellite DNA sequences, CaSat1 and CaSat2. While CaSat1 localizes to subtelomeric regions of the two largest chromosomes, CaSat2 is located in centromeric regions of all eight chromosomes (Staginnus et al., 1999). The attempts to identify the remaining chromosomes, which included fluorescence *in situ* hybridization (FISH) with probes for some microsatellites and retrotransposons, were largely unsuccessful (Sharma et al., 1995; Gortner et al., 1998; Staginnus et al., 1999, 2010).

Conventional microscopic identification of all chromosomes of chickpea using a variety of banding techniques and fluorescence staining with chromomycin A3 and 4′,6-diamidino-2-phenylidole (DAPI) enable unambiguous identification of the two largest chromosomes and also the smallest chromosome. The remaining chickpea chromosomes are similar in size and metacentric, which hampers discrimination of individual arms (Ohri and Pal, 1991; Begum and Alam, 2016a,b). The application of FISH with a set of traditionally used probes, which included genes for 5S and 45S rRNA, two satellite DNA sequences CaSat1 and CaSat2, and probes developed from transposable element sequences (e.g., CaRep repeats) enabled unambiguous identification of the two largest chromosomes (with hybridization signals of rDNA and CaSat1) and an intermediate-sized chromosome with another hybridization signal of 5S rDNA (Galasso et al., 1996; Staginnus et al., 1999, 2001, 2010). Fluorescence *in situ* hybridization probes for satellite repeat CaSat2 localized to centromeric regions of all chromosomes (Staginnus et al., 1999), and the probes developed from transposable elements were dispersed across all chromosome arms (Staginnus et al., 2001, 2010).

The identification of all eight chromosomes of chickpea was achieved only after they were classified according to relative DNA content and sorted using flow cytometry. Initially, Vláčilová et al. (2002) discriminated five peaks on a flow karyotype of kabuli type of chickpea and anchored one of the chromosomes—the smallest in the set—to genetic linkage group 8 using polymerase chain reaction (PCR) with primers for sequence-tagged microsatellite site markers. Using FISH, the authors revealed the presence of large interstitial blocks of telomeric repeats (localized on the same arm as 45S rDNA) on chromosomes labeled A and B, and confirmed the location of 5S rDNA on the large chromosome B and on the intermediate-sized chromosome G. In a follow-up study, Zatloukalová et al. (2011) created the first complete molecular karyotype of desi-type chickpea after combining chromosome flow sorting and developing new probes for FISH. These comprised five unique BAC clones (10I13, 14M02, 17N07, 11K07, and 15M06), which hybridized to single genomic loci. Using the single-copy BAC clones and the previously used probes for FISH on flow-sorted chromosomes, the authors succeeded in identifying all eight chromosomes. However, three out of the eight chromosomes were identified only after flow sorting (Zatloukalová et al., 2011; Karafiátová et al., 2017). The persistent bottleneck was the shortage of probes for FISH to obtain chromosome-specific labeling patterns.

Recently, Han et al. (2015) developed oligopainting FISH, which has been successfully applied in a variety of plant species to create molecular karyotypes, identify individual chromosomes, and reveal large-scale chromosomal translocations (e.g., Braz et al., 2018; Xin et al., 2018; Šimoníková et al., 2019, 2020). The approach is based on *in silico* identification of thousands of unique short oligomers (∼ 50-nt long) in a genome sequence (typically a chromosome-scale reference genome) and their synthesis and use as probes for FISH after direct or indirect fluorescent labeling (reviewed in Jiang, 2019). The oligopainting probes can be designed to cover entire chromosomes or only chromosome arms to label multiple regions of multiple chromosomes and obtain a chromosome-specific FISH pattern. The latter approach was termed the oligo-FISH barcode system (Braz et al., 2018) and is based on the production of two different oligomer libraries, which facilitate unambiguous identification of all chromosomes of a species (e.g., Braz et al., 2020; Chen et al., 2020; Liu et al., 2020).

This study aimed at the creation of a universal oligo-FISH barcoding system in chickpea to (1) establish molecular karyotypes of desi and kabuli chickpea; (2) anchor draft genome sequences (pseudomolecules) to chromosomes *in situ*; (3) compare the structure of colinear chromosomes and identify large chromosome structural changes between desi and kabuli genotypes; and (4) identify misassembled regions *in situ*.

## Materials and Methods

### Plant Material and Chromosome Spreads Preparation

Seeds of two different accessions of chickpea (*Cicer arientinum* L., 2*n* = 2*x* = 16), ICC 1882 (desi type) and CDC Frontier (kabuli type), were obtained from the International Crops Research Institute for the Semi-Arid Tropics (ICRISAT, Patancheru, India). Seed germination, cell cycle synchronization, and metaphase accumulation in root meristem cells were performed according to Vláčilová et al. (2002). Synchronized roots were fixed for 20 min in 2% (v/v) formaldehyde in Tris buffer at 4°C. After three 5-min washes in Tris buffer (Vláčilová et al., 2002) the roots were used to prepare protoplast suspension following Doležel et al. (1998), with minor modifications. Segments of root meristems were excised and digested in a mixture of 2% (w/v) cellulase, 2% (w/v) pectinase, and 1% (w/v) cytohelicase in 75 mM KCl and 7.5 mM ethylenediaminetetraacetic acid (EDTA) (pH 4), for 90 min at 30°C. The crude suspension was filtered through 150-μm nylon mesh, pelleted by centrifugation (1,000 rpm, 5 min, 4°C), and washed in 75 mM KCl and 7.5 mM EDTA buffer. Pelleted protoplasts were diluted in 50 μl of 70% ethanol, and stored at −20°C for further use. Mitotic metaphase chromosome spreads were prepared using the dropping technique of Doležel et al. (1998). Air-dried slides were postfixed in 4% (v/v) formaldehyde made in 2 × saline-sodium citrate (SSC) (10 min at room temperature), washed in 2 × SSC for 2 × 5 min, and dehydrated using ethanol series and used for FISH. Mitotic metaphase spreads of both morphotypes were counterstained with DAPI and mounted in Vectashield antifade mounting medium (Vector Laboratories, Burlingame, California, United States), and used to determine chromosome and chromosome-arm length to create schematic karyotypes (idiograms). The measurements were conducted using ISIS software (Metasystems, Altlussheim, Germany) in ten complete metaphase plates of each chickpea genotype (Supplementary Table 1).

### Oligo-Fluorescence *in situ* Hybridization Probe Design and *in silico* Mapping

**FIGURE 1:**
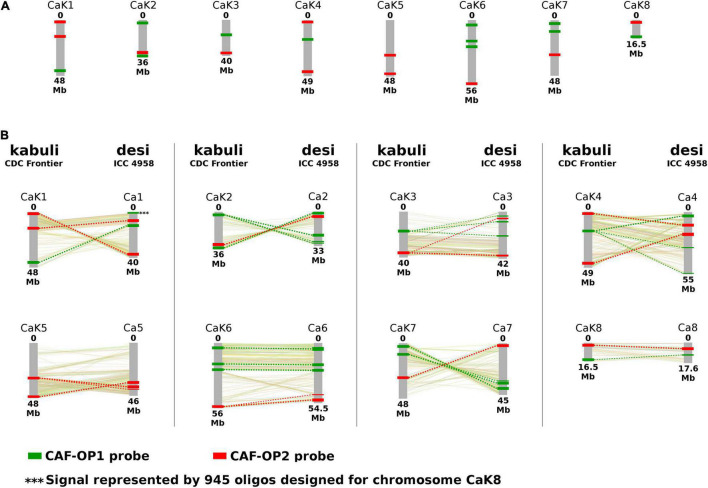
Location of oligopainting barcodes CAF-OP1 (green) and CAF-OP2 (red). **(A)** Oligopainting barcodes were identified and selected by Chorus software on a reference genome assembly of *Cicer arietinum* CDC Frontier (kabuli type). **(B)** Schematic comparison of *in silico* predicted positions of oligopainting barcodes on a reference genome of *C. arietinum* CDC Frontier (kabuli type) and *C. arietinum* ICC 1882 (desi type). A collinearity between kabuli and desi pseudomolecules is shown based on the results of Parween et al. (2015). Chromosome nomenclature corresponds to the pseudomolecules of Varshney et al. (2013) and Parween et al. (2015).

Oligonucleotides suitable for probe design were identified using the Chorus software.^1^ To develop probes for chromosome barcoding, we selected ∼ 500-kb-long regions on chromosomes of CDC Frontier (kabuli type; Varshney et al., 2013) which shared high homology with the regions on collinear chromosomes of desi accession ICC 4958 (Parween et al., 2015). The draft genome sequence of CDC Frontier was used to design two sets of 20,000 oligomers (45-nt) covering specific regions on chromosomes (Figure 1). The oligomers were synthesized as immortal libraries by Arbor Biosciences (Ann Arbor, Michigan, United States) and then labeled either by digoxigenin or biotin (Eurofins Genomics, Ebersberg, Germany) according to Han et al. (2015).

To compare the predicted locations of oligomers on pseudomolecules of kabuli and desi types, sequences representing bulked oligoprobes were mapped to their genome assemblies by BWA v0.7.17 (Li and Durbin, 2010) and visualized along the pseudomolecules using BEDTools v2.27.1 (Quinlan and Hall, 2010). An additional painting probe was designed to confirm the translocated region (0–10 Mb) on chromosome 7 of desi type of *C. arietinum* (genotype ICC 1882), which was identified during this work. The oligomers were synthesized as immortal libraries by Arbor Biosciences (Ann Arbor, Michigan, United States) and labeled by Cy5 (Eurofins Genomics, Ebersberg, Germany) following Han et al. (2015).

### Illumina Sequencing and Assembly of Bacterial Artificial Chromosome Clones

Three BAC clones (10I13, 11K07, and 14M02) which localized to singe loci on chromosomes of desi accession WR315 (Zatloukalová et al., 2011) and one additional BAC 05E03, which we identified later, were sequenced using Illumina. DNA of the BACs was extracted using the Qiagen Plasmid Midi Kit (Qiagen), sheared by Bioruptor Plus (Diagenode, Liege, Belgium) to achieve an insert size of about 800 bp. Sequencing libraries were prepared from 2 μg of fragmented DNA using TruSeq^®^ DNA PCR-free kit (Illumina) and sequenced on the MiSeq Illumina platform with reads length 2 × 300 bp to achieve a minimal sequence depth of 100 ×. Illumina sequences were processed using homemade perl script combining the assembly processes using MaSuRCA (Zimin et al., 2013) and identification of vector sequences flanking the chickpea assembled contigs/scaffolds. Final scaffolds were then used to map onto desi and kabuli pseudomolecules using MegaBLAST (Altschul et al., 1990) to determine their relative position concerning oligopainting probes.

### Preparation of Other Probes for Fluorescence *in situ* Hybridization

45S rDNA probe was prepared from a clone pTA71 (Gerlach and Bedbrook, 1979) by nick translation (Roche Applied Science, Penzberg, Germany) using aminoallyl-dUTP-Cy5 (Jena Biosciences, Jena, Germany) according to the instructions of the manufacturer. A probe for 5S rDNA was prepared according to Zatloukalová et al. (2011) and labeled by aminoallyl-dUTP-Cy5 (Jena Biosciences). A probe for telomere repeat ([CCCTAAA]_4_) was synthesized as a 5′-end-labeled oligomer by Cy5 fluorochrome (Integrated DNA Technologies, Inc., Coralville, IA, United States). DNA of BAC clones 14M02, 05E03, 11K07, and 10I13 (Zatloukalová et al., 2011) was isolated by alkaline lysis and labeled by nick translation (Roche Applied Science) using aminoallyl-dUTP-Cy5 (Jena Biosciences) according to the instructions of the manufacturer.

### Fluorescence *in situ* Hybridization

Hybridization mix containing 50% (v/v) formaldehyde, 10% (w/v) dextran sulfate in 5 × SSC, and 200 ng of labeled probes was applied onto the slides and denatured for 2 min 15 s at 80°C. Hybridization was carried out overnight at 37°C in a humid chamber. After the hybridization, the stringent wash was performed in 0.1 × SSC (2 mmol/l MgCl_2_ and 0.1% Triton X-100) (Schwarzacher and Heslop-Harrison, 2000) for 10 min at 42°C according to Vláčilová et al. (2002). Indirectly labeled probes were detected using antidigoxigenin-FITC (Roche Applied Science) and Streptavidin-Cy3 (ThermoFisher Scientific/Invitrogen, Waltham, United States). The preparations were counterstained with DAPI and mounted in Vectashield antifade mounting medium (Vector Laboratories). Images of metaphase plates were acquired with Axio Imager Z.2 Zeiss microscope (Zeiss, Oberkochen, Germany) equipped with Cool Cube 1 camera (Metasystems) and appropriate optical filters. The captured images were processed with ISIS software (Metasystems). Karyotypes were constructed after the analysis of at least twenty complete metaphase plates and their images were created using Adobe Photoshop CS5 (version 12.0).

## Results

### Development of Barcoding Probes for Chromosome Identification

We have developed two bulked oligopainting probes for chromosome identification in *C. arietinum*. The probes comprise 18,677 and 19,917 oligonucleotides (45-nt), which were identified in the reference genome sequence of kabuli-type CDC Frontier (Varshney et al., 2013). The probe CAF-OP1 (green signals) covers 11 different regions on seven chickpea pseudomolecules, while the probe CAF-OP2 (red signals) covers the other 11 regions on eight chickpea pseudomolecules (Figure 1A and Supplementary Table 2). The size of each of the 11 chromosome regions was ∼500 kb on the kabuli pseudomolecule and it was covered by 1,202–2,108 oligonucleotides. Oligo-FISH barcode libraries, hereafter called painting probes, were designed to achieve a density of 2.00–4.20 oligos per 1 kb in the region of interest (Supplementary Table 2), to ensure good visibility of hybridization signals after FISH.

To confirm the suitability of the painting probes for chromosome identification in desi chickpea, we mapped the probe sequences *in silico* to the reference genome of the ICC 4958 accession (Parween et al., 2015). The results showed that most of the kabuli-derived oligomers mapped to collinear chromosome regions (Figure 1B). However, some exceptions were revealed for the CAF-OP1 painting probe specific to kabuli chromosome 8 (1,656 oligonucleotides), which showed homology to two different genome regions in the desi accession ICC 4958. The first region of the CAF-OP1 painting probe specific to kabuli chromosome 8 (∼ 150 kb containing 557 oligomers) showed homology to ∼ 200-kb-long region on the collinear chromosome 8 in the desi accession ICC 4958. The second region of the CAF-OP1 painting probe specific to kabuli chromosome 8 (∼ 200 kb containing 945 oligomers) was homologous to the ∼ 300-kb-long region on chromosome 1 in the desi accession (Figure 1B). *In silico* mapping of oligomers designed for kabuli type of *C. arietinum* genome to the desi genotype revealed other short regions (up to 100 kb) in the desi genome covered by the low number of kabuli-specific oligomers (Figure 1).

### Chromosome Identification and Karyotype Development

**FIGURE 2:**
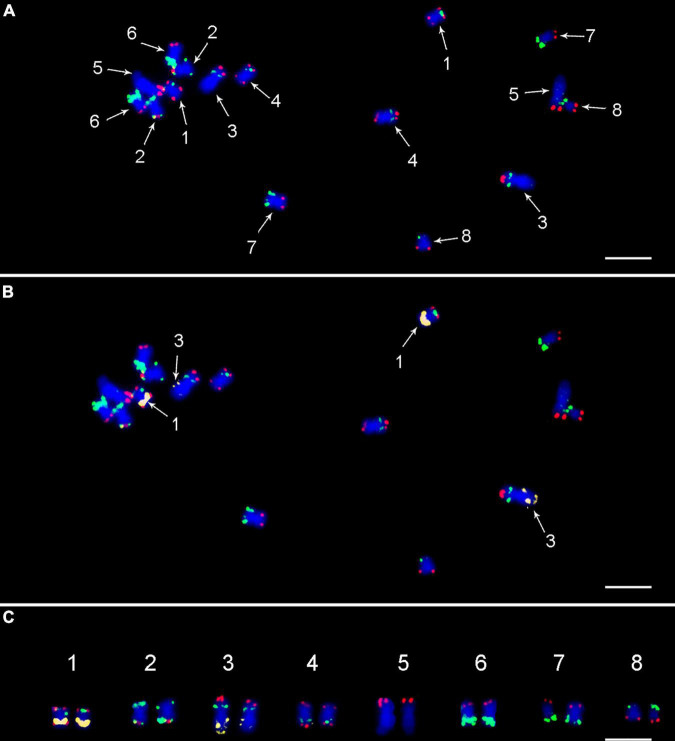
FISH on mitotic metaphase plate of *Cicer arietinum* CDC Frontier (kabuli type) using **(A)** painting probes CAF-OP1 (green) and CAF-OP2 (red); **(B)** combination of painting probes and 5S rDNA probe (yellow), which was used to unambiguously distinguish chromosomes 1 and 3. **(C)** Molecular karyotype of CDC Frontier genotype (kabuli type). Chromosomes were counterstained with DAPI (blue). Bar = 3 μm.

**FIGURE 3:**
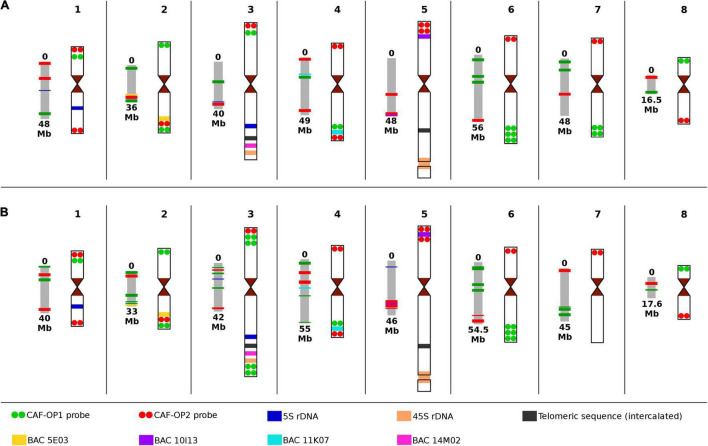
Scheme of pseudomolecules with corresponding chromosome idiograms of the two types of chickpea with the positions of all cytogenetic landmarks and painting probes used in this study. **(A)** *Cicer arietinum* CDC Frontier, kabuli type; and **(B)** C. *arietinum* ICC 1882, desi type. Chromosome nomenclature corresponds to the pseudomolecules of Varshney et al. (2013) and Parween et al. (2015).

To validate the suitability of the CAF-OP1 and CAF-OP2 painting probes for chromosome identification *in situ*, they were used for FISH on mitotic metaphase spreads of kabuli-type CDC Frontier, whose reference genome was used to design the painting probes. The positions of the majority of FISH signals were in accordance with *in silico* predictions, the exception being chromosome 1 in kabuli type, where the positions of two barcodes were rearranged. This, however, led to the same labeling pattern on chromosomes 1 and 4 (Figure 2). Thus for unambiguous chromosome identification, we colocalized CAF-OP1 and CAF-OP2 painting probes with previously developed cytogenetic landmarks, namely, rDNA sequences, telomeric sequences, and four selected BAC clones (Supplementary Figure 2). As expected, 5S rRNA genes localized to the long arms of chromosomes 1 and 3 in the kabuli genome (Figure 2). FISH with the probe for 45S rDNA resulted in hybridization signals in the nucleolus organizer region on chromosome 5, with an additional signal in the subtelomeric region of the long arm of chromosome 3 (Figure 3A and Supplementary Figure 2E). Interstitial signals were also observed after FISH with telomere DNA repeat and were visible on the long arm of chromosomes 3 and 5 (Figure 3A and Supplementary Figure 2C). As this has been the first study in which the pseudomolecules of chickpea genome assembly were anchored to chromosomes *in situ*, we wanted to create more complex karyotypes. To achieve this, we colocalized the painting probes with four chickpea BAC clones which hybridized into single loci onto specific chromosomes.

Bacterial artificial chromosome (BAC) clones 05E03 and 11K07 were localized to the long arm of chromosomes 2 and 4 in kabuli and the hybridization signal positions were in accordance with *in silico* predictions (Figure 3 and Supplementary Figure 2). BAC 10I13, which localized to the short arm of chromosome 5, provided a hybridization signal behind the red signals of the CAF-OP2 painting probe, which did not correspond to the *in silico* prediction. Thus, this region on the kabuli pseudomolecule seems to be wrongly assembled—inverted as compared to the physical localization on the chromosome (Figure 3 and Supplementary Figure 2). Finally, BAC 14M02 localized to the long arm of chromosome 3 in the kabuli genome (Supplementary Figure 2). However, the position of the BAC 14M02 sequence on the pseudomolecules of kabuli could not be established, most probably due to the absence of this region in the genome assembly.

After establishing the karyotype of kabuli-type CDC Frontier, we conducted FISH with the same set of probes on chromosomes of desi accession ICC 1882. The same hybridization pattern of CAF-OP1 (green) and CAF-OP2 (red) painting probes was observed on chromosomes 1, 2, 4, 5, 6, and 8 (Figure 3 and Supplementary Figure 1). However, different barcode labeling patterns were observed on chromosomes 3 and 7 as compared to the kabuli type. This pattern also differed from that predicted *in silico* in the desi genome (Figure 1). When compared to the kabuli type, chromosome 3 of the desi accession contained two additional green bands in the subtelomeric region of its long arm, while chromosome 7 missed two green bands on its long arm (Figure 3 and Supplementary Figure 3). One additional green painting signal was also observed on the short arm of desi chromosome 3 (Figure 3 and Supplementary Figure 3). This observation indicated the presence of chromosome translocation in the desi accession ICC 1882.

Integrated karyotyping of desi type of chickpea using a combination of painting probes and other cytogenetic landmarks showed additional rearranged regions on desi chromosomes compared to kabuli. The probes for 45S rDNA, interstitial telomere repeats, and the three BAC clones (05E03, 11K07, and 14M02) localized to similar positions on the same chromosomes in desi as on chromosomes of kabuli CDC Frontier (Figure 3 and Supplementary Figure 3). However, the difference was observed for BAC 10I13 that localized between red signals of the CAF-OP2 painting probe on the short arm of chromosome 5 (Figure 3 and Supplementary Figure 3D), indicating intrachromosomal inversion in desi.

### Validation of Chromosome Translocation in Desi Accession ICC 1882

**FIGURE 4:**
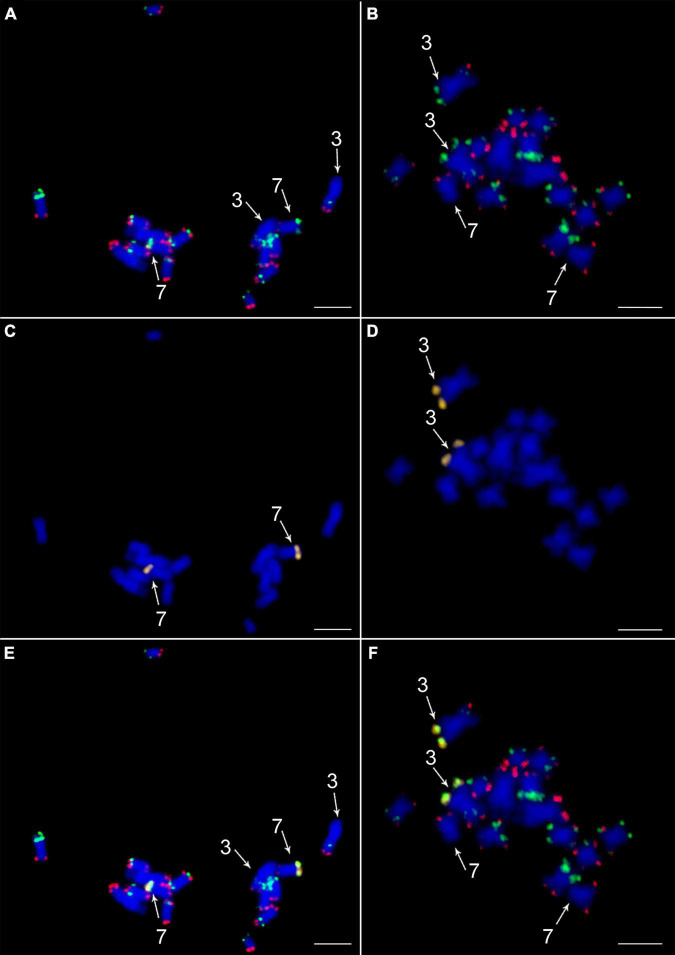
Validation of translocation of a telomeric part of long arm of chromosome 7 to the long arm of chromosome 3 in desi type of *Cicer arietinum* ICC 1882. *In situ* localization of the two painting probes CAF-OP1 (green) in CAF-OP2 (red) on metaphase plates of **(A)** *C. arietinum* CDC Frontier (kabuli) and **(B)** *C. arietinum* ICC 1882 (desi). Localization of a probe specific to the translocated part of chromosome 7 (yellow) in the **(C)** kabuli and **(D)** desi genotypes. Colocalization of CAF-OP1 (green), CAF-OP2 (red), and a probe specific to the translocated part of chromosome 7 (yellow) on mitotic metaphase plates of the **(E)** kabuli and **(F)** desi types. Chromosomes were counterstained with DAPI (blue). Chromosomes 3 and 7 are marked by arrows. Bar = 3 μm.

To confirm a translocation of the distal region of the long arm of chromosome 7 to the long arm of chromosome 3 in desi, we designed an additional oligonucleotide painting probe specific to the putative translocated region on chromosome 7. It was designed to cover the translocated region and corresponded to the 10-Mb-long region on pseudomolecule Ca7 of the desi reference genome (Parween et al., 2015). Fluorescence *in situ* hybridization with this probe confirmed the *in silico* prediction as the hybridization signal was observed in the subtelomeric region of the long arm of desi chromosome 3 (Figure 4). A difference in structure and organization of chromosome 3 between desi and kabuli genotypes was also observed for the short arm of chromosome 3, which in desi type contained one additional green oligopainting signal. This observation agreed with *in silico* prediction on desi reference genome sequence (Parween et al., 2015).

Another putative chromosome rearrangement in desi chickpea concerning kabuli was identified *in silico* by mapping CAF-OP1 oligomers specific to kabuli chromosome 8. These oligomers mapped *in silico* to two chromosomes (1 and 8) in desi reference genome sequence. However, the absence of additional green bands on desi chromosome 1 (Figure 3 and Supplementary Figure 3) after FISH with the painting CAF-OP1 and CAF-OP2 probes did not confirm this putative chromosome rearrangement in desi accession ICC 1882.

### Comparison of Predicted *in silico* and Physical Position of Cytogenetic Landmarks

At the cytogenetic level, the general structure and long-range organization of a majority of chickpea chromosomes shared a high level of collinearity. The position of signals of painting probes and other cytogenetic landmarks was similar on kabuli and desi chromosomes. The only exception was chromosomes 7 and 3, which differed by a telomeric translocation in the desi genome (Figure 3). The short arm of chromosome 3 in desi contained also an additional green painting signal. As the painting probes represented a group of barcodes designed to label different regions in the genome, we cannot exclude a possibility that this addition signal indicated a duplication. The additional short inverted region was detected on the short arm of chromosome 5 in desi, where the position of red painting signals and BAC 10I13 was rearranged compared to kabuli (Figure 3).

In contrast to the highly conserved physical organization of the landmarks along the chromosomes between kabuli and desi types of chickpea at cytogenetic level, *in silico* mapping of the nucleotide sequences onto pseudomolecules showed a remarkable difference, especially in desi. However, it should be noted that it was not possible to map all sequences used as cytogenetic landmarks to the pseudomolecules. The assemblies do not contain regions with 45S rDNA sequences, telomeric sequences—even those which are intercalated as large clusters on the long arms of chromosomes 3 and 5, and BAC 14M02.

In kabuli, the order of *in silico* mapped landmarks on the pseudomolecules mostly agreed with their physical location and the order as determined by FISH. The only exception was the location of 5S rRNA genes on pseudomolecule CaK3, which did not correspond with its physical location on chromosome 3 of kabuli (Figure 3).

The situation was different in desi chickpea. As already mentioned, discrepancies were found between the predicted positions of oligo-FISH barcodes on pseudomolecules (as compared to kabuli) and their physical location on chromosomes (Figures 1, 3). Different *in silico* positions were observed also for BAC clones 05E03 and 11K07. Although they were identified on the expected pseudomolecules, their position was not congruent with their location on chromosomes (Figure 3). The discrepancies between *in silico* and physical location were also observed for 5S rDNA sequences, which localized by FISH to the long arm of chromosome 1 and the long arm of chromosome 3. However, pseudomolecule Ca1 did not contain any 5S rDNA sequence and the position of 5S rDNA on pseudomolecule Ca3 was different as compared to its physical location (Figure 3). On the other hand, pseudomolecule 5 contains a region with 5S rDNA sequences.

## Discussion

Similar to many other plant species, namely, some important crops, the identification of chickpea chromosomes *in situ*, comparative karyotyping, and the analysis of the behavior of individual chromosomes during meiosis remain a major challenge (Vláčilová et al., 2002; Zatloukalová et al., 2011; Karafiátová et al., 2017). This is a consequence of a shortage of probes suitable for FISH that would give chromosome-specific labeling patterns.

The recent development of oligopainting FISH provided an opportunity to establish cytogenetic barcoding systems for rapid characterization of plant karyotypes (Braz et al., 2018, 2020; Chen et al., 2020; Liu et al., 2020). In this study, we developed an oligo-FISH barcode system for kabuli-type chickpea, CDC Frontier (Varshney et al., 2013). Each probe was designed to loci highly homologous to the reference genome sequence of a desi type (ICC 4958) to enable their use in comparative karyotype analysis of both cultivated types of chickpea. The reference genome sequences of the chickpea kabuli and desi types represent about 25%–58% of the estimated chromosome length (Karafiátová et al., 2017), and the positions of centromeric regions remain unknown. This is probably due to the presence of DNA repeats, including the large blocks of satellite DNA (CaSat2) (Staginnus et al., 1999; Zatloukalová et al., 2011), which hampered the creation of chromosome-scale assemblies.

We performed oligo-FISH in kabuli type of chickpea using the same genotype from which reference genome assembly was produced (Varshney et al., 2013). This made it possible to validate the method and compare *in silico* location of the painting probes with their physical chromosomal positions. The positions of the majority of painting barcodes agreed with *in silico* predictions except for chromosome 1, where the positions of two barcodes were rearranged. This observation pointed to some incorrectly assembled regions of the pseudomolecule of kabuli chickpea. A combination of painting probes with probes for 5S rDNA and BAC 10I13 revealed other incorrectly assembled, most probably inverted regions on kabuli chromosomes 3 and 5 (Figure 3). More striking differences between the predicted *in silico* organization of painting probes and cytogenetic landmarks were observed for a majority of chromosomes of desi (Figure 3) which may reflect different strategies employed to create pseudomolecules of kabuli and desi genomes (Ruperao et al., 2014; Parween et al., 2015).

In contrast to the *in silico* comparison of the kabuli and desi genome sequences (Parween et al., 2015), which indicated differences in long-range chromosome organization of all chickpea chromosomes, our results suggest that this apparent structural variability, at least in some cases, could be due to incorrect scaffolding. If so, this could be improved by long-read sequencing technologies in combination with the Hi-C method and/or Bionano optical mapping, as done in other plant species with complex genomes (Belser et al., 2018; Deschamps et al., 2018; Wang et al., 2020; Bredeson et al., 2021).

Our comparative cytogenetic analysis of a chickpea desi-type accession (ICC 1882) and kabuli-type accession (CDC Frontier) indicated general chromosome collinearity between both types. These results imply a high level of genome similarity between kabuli and desi cultivars and confirm their close phylogenetic relationships (Thudi et al., 2011; Roorkiwal et al., 2014; Gupta et al., 2017). However, a difference was observed for the signals of the barcode CAF-OP2 (red signals) and BAC clone 10I13 on the short arm of chromosome 5 of desi type as compared to kabuli, indicating intrachromosomal inversion. Similarly, the short arm of chromosome 3 in desi type contained one additional green signal as compared to kabuli. If the *in silico* prediction of the oligobarcoding pattern on the desi genome sequence is correct, this observation points to insertion in the desi genome region relative to the kabuli genome. The most striking chromosome structural variability identified in this work involved long arms of chromosomes 7 and 3, indicating the presence of a translocation event between the two chromosomes in the desi type of chickpea. This translocation was confirmed by FISH with a specific oligopainting probe, which covered the putative translocated region.

Oligopainting FISH was found useful to identify large chromosome translocations in a range of closely related species (Braz et al., 2018; Hou et al., 2018; Albert et al., 2019; Šimoníková et al., 2020; do Vale Martins et al., 2021). However, we were not able to ascertain if the translocation between the long arm of chromosomes 3 and 7 was reciprocal. A comparison of the current genome assemblies of CDC Frontier and ICC 4958 accessions did not reveal this translocation event (Parween et al., 2015). To clarify this issue and to locate the translocation breakpoint, an improved chromosome-scale genome assembly will be needed. It should be pointed out that we used a different genotype of desi (ICC 1882) for cytogenetic mapping than the one used for genome assembly. Thus, although the translocation could be the result of incorrectly assembled regions on pseudomolecules of desi-type chickpea, it may also be genotype-specific. To clarify this, more kabuli and desi types accessions should be analyzed by oligo-FISH barcoding. In any case, our data indicate that kabuli and desi genome assemblies may contain incorrectly assembled regions and that the oligopainting FISH is one of the possible approaches to identify them.

## Data Availability Statement

The datasets presented in this study can be found in online repositories. The names of the repository/repositories and accession number(s) can be found below: https://datadryad.org/stash, https://doi.org/10.5061/dryad.66t1g1k32.

## Author Contributions

EH conceived the study and performed bioinformatic analyses. AD, LS, DŠ, KH, and MK conducted the study and processed the data. RKV contributed in providing material, data analysis, and interpretation. AD, JD, and EH wrote the manuscript. All authors discussed the results, contributed to manuscript writing, and read and approved the final manuscript.

## Conflict of Interest

The authors declare that the research was conducted in the absence of any commercial or financial relationships that could be construed as a potential conflict of interest.

## Publisher’s Note

All claims expressed in this article are solely those of the authors and do not necessarily represent those of their affiliated organizations, or those of the publisher, the editors and the reviewers. Any product that may be evaluated in this article, or claim that may be made by its manufacturer, is not guaranteed or endorsed by the publisher.

## Footnotes

1
https://github.com/forrestzhang/Chorus

## Supplementary Material

Supplementary Figure 1 ISH on mitotic metaphase plate of *C. arietinum* ICC 1882 (desi type) using **(A)** painting probes CAF-OP1 (green) and CAF-OP2 (red); **(B)** combination of the painting probes and 5S rDNA probe (yellow); and **(C)** molecular karyotype of ICC 1882 genotype (desi type). Chromosomes were counterstained with DAPI (blue). Bar = 3 μm.

Supplementary Figure 2 Integration of oligopainting probe CAF-OP1 (green) and CAF-OP2 (red) with the extant cytogenetics landmarks (yellow) on mitotic metaphase chromosomes of *C. arietinum* CDC Frontier (kabuli): **(A)** 5S rDNA; **(B)** BAC 05E03; **(C)** telomeric sequence [TTTAGGG]*_*n*_*; **(D)** BAC 14M02; **(E)** 45S rDNA; **(F)** BAC 10I13; and **(G)** BAC 11K07, on mitotic metaphase chromosomes of *C. arietinum* CDC Frontier (kabuli). Chromosomes were counterstained with DAPI (blue). Arrows indicate positions of BAC clones or rRNA probes. Bar = 3 μm.

Supplementary Figure 3 Integration of oligopainting probe CAF-OP1 (green) and CAF-OP2 (red) with the extant cytogenetics landmarks (yellow) on mitotic metaphase plates of *C. arietinum* ICC 1882 (desi): **(A)** 5S rDNA; **(B)** BAC 11K07; **(C)** 45S rDNA; **(D)** BAC 10I13; and **(F)** BAC 5E03 (yellow). **(E)** Localization of oligopainting probe CAF-OP1 (green) with telomeric sequence (red) and BAC 10I13. **(G)** Colocalization of oligopainting probe CAF-OP1 (green) with telomeric sequence (red) and BAC 14M02 (yellow). Chromosomes were counterstained with DAPI (blue). Arrows indicate positions of BAC clones or rRNA probes. Bar = 3 μm.

## References

[B1] Abbo S. Berger J. Turner N. C. (2003). Evolution of cultivated chickpea: four bottlenecks limit diversity and constrain adaptation. *Funct. Plant Biol.* 30 1081–1087. 10.1071/FP03084 32689090

[B2] Albert P. S. Zhang T. Semrau K. Rouillard J. M. Kao Y. H. Wang C. J. R. (2019). Whole-chromosome paints in maize reveal rearrangements, nuclear domains, and chromosomal relationships. *Proc. Natl. Acad. Sci. U.S.A.* 116 1679–1685. 10.1073/pnas.1813957116 30655344 PMC6358699

[B3] Altschul S. F. Gish W. Miller W. Myers E. W. Lipman D. J. (1990). Basic local alignment search tool. *J. Mol. Biol.* 215 403–410. 10.1016/S0022-2836(05)80360-2 2231712

[B4] Arumuganathan K. Earle E. D. (1991). Nuclear DNA content of some important plant species. *Plant Mol. Biol. Rep.* 9 208–218. 10.1007/BF02672069

[B5] Barmukh R. Roorkiwal M. Jaba J. Chitikineni A. Mishra S. P. Sagurthi S. R. (2021). Development of a dense genetic map and QTL analysis for pod borer Helicoverpa armigera (Hübner) resistance component traits in chickpea (*Cicer arietinum* L.). *Plant Genome* 14:e20071. 10.1002/tpg2.20071 33289349 PMC12807004

[B6] Begum K. N. Alam S. S. (2016a). Karyomorphological analysis with differential staining of nine *Cicer arietinum* L. varieties. *Bangladesh J. Bot.* 45 327–334.

[B7] Begum K. N. Alam S. S. (2016b). Differential fluorescent banding in nine varieties of *Cicer arietinum* L. *Cytologia* 81 383–387. 10.1508/cytologia.81.383

[B8] Belser C. Istace B. Denis E. Dubarry M. Baurens F. C. Falentin C. (2018). Chromosome-scale assemblies of plant genomes using nanopore long reads and optical maps. *Nat. Plants* 4 879–887. 10.1038/s41477-018-0289-4 30390080

[B9] Braz G. T. do Vale Martins L. Zhang T. Albert P. S. Birchler J. A. Jiang J. (2020). A universal chromosome identification system for maize and wild Zea species. *Chromosome Res.* 28 183–194. 10.1007/s10577-020-09630-5 32219602

[B10] Braz G. T. He L. Zhao H. Zhang T. Semrau K. Rouillard J. M. (2018). Comparative oligo-FISH mapping: an efficient and powerful methodology to reveal karyotypic and chromosomal evolution. *Genetics* 208 513–523. 10.1534/genetics.117.300344 29242292 PMC5788518

[B11] Bredeson J. V. Lyons J. B. Oniyinde I. O. Okereke N. R. Kolade O. Nnabue I. (2021). High contiguity de novo genome sequence of Trifoliate yam (*Dioscorea dumetorum*) using long read sequencing. *bioRxiv* [Preprint] bioRxiv 2021.04.14.439117, 10.1101/2021.04.14.439117

[B12] Chen L. Su D. Sun J. Li Z. Han Y. (2020). Development of a set of chromosome-specific oligonucleotide markers and karyotype analysis in the Japanese morning glory Ipomoea nil. *Sci. Hortic.* 273:109633. 10.1016/j.scienta.2020.109633

[B13] Deokar A. Sagi M. Tar’an B. (2019). Genome-wide SNP discovery for development of high-density genetic map and QTL mapping of ascochyta blight resistance in chickpea (*Cicer arietinum* L.). *Theor. Appl. Genet.* 132 1861–1872. 10.1007/s00122-019-03322-3 30879097 PMC6531409

[B14] Deschamps S. Zhang Y. Llaca V. Ye L. Sanyal A. King M. (2018). Chromosome-scale assembly of the sorghum genome using nanopore sequencing and optical mapping. *Nat. Commun.* 9:4844. 10.1038/s41467-018-07271-1 30451840 PMC6242865

[B15] do Vale Martins L. de Oliveira Bustamante F. da Silva Oliveira A. R. da Costa A. F. de Lima Feitoza L. Liang Q. (2021). BAC- and oligo-FISH mapping reveals chromosome evolution among *Vigna angulatris*, *V. enguiculata*, and *Phaseolus vulgaris*. *Chromosoma* 130 133–147. 10.1007/s00412-021-00758-9 33909141

[B16] Doležel J. Doleželová M. Roux N. Van den houwe I. (1998). A novel method to prepare slides for high resolution chromosome studies in *Musa* spp. *Infomusa* 7 3–4.

[B17] Galasso I. Pignone D. Frediani M. Maggiani M. Cremonini R. (1996). Chromatin characterization by banding techniques, in situ hybridization, and nuclear DNA content in *Cicer* L. (Leguminosae). *Genome* 39 258–265. 10.1139/g96-035 18469891

[B18] Gaur R. Jeena G. Shah N. Gupta S. Pradhan S. Tyagi A. K. (2015). High density linkage mapping of genomic and transcriptomic SNPs for synteny analysis and anchoring the genome sequence of chickpea. *Sci. Rep.* 5:13387. 10.1038/srep13387 26303721 PMC4548218

[B19] Gerlach W. L. Bedbrook J. R. (1979). Cloning and characterization of ribosomal RNA genes from wheat and barley. *Nucleic Acids Res.* 7 1869–1885. 10.1093/nar/7.7.1869 537913 PMC342353

[B20] Gortner G. Nenno M. Weising K. Zink D. Nagl W. Kahl G. (1998). Chromosomal localization and distribution of simple sequence repeat and the *Arabidopsis*-type telomere sequence in the genome of *Cicer arietinum* L. *Chromosome Res.* 6 97–104. 10.1023/A:1009282828236 9543012

[B21] Gupta S. Nawaz K. Parween S. Roy R. Sahu K. Pole A. K. (2017). Draft genome sequence of *Cicer reticulatum* L., the wild progenitor of chickpea provides a resource for agronomic trait improvement. *DNA Res.* 24 1–10. 10.1093/dnares/dsw042 27567261 PMC5381347

[B22] Han Y. Zhang T. Thammapichai P. Weng Y. Jiang J. (2015). Chromosome-specific painting in *Cucumis* species using bulked oligonucleotides. *Genetics.* 200 771–779. 10.1534/genetics.115.177642 25971668 PMC4512542

[B23] Hiremath P. J. Kumar A. Penmetsa R. V. Farmer A. Schlueter J. A. Chamarthi S. (2012). Large-scale development of cost-effective SNP marker assays for diversity assessment and genetic mapping in chickpea and comparative mapping in legumes. *Plant Biotechnol. J.* 10 716–732. 10.1111/j.1467-7652.2012.00710.x 22703242 PMC3465799

[B24] Hou L. Xu M. Zhang T. Xu Z. Wang W. Zhang J. (2018). Chromosome painting and its applications in cultivated and wild rice. *BMC Plant Biol.* 18:110. 10.1186/s12870-018-1325-2 29879904 PMC5991451

[B25] Jain M. Misra G. Patel R. K. Priya P. Jhanwar S. Khan A. W. (2013). A draft genome sequence of the pulse crop chickpea (*Cicer arietinum* L.). *Plant J.* 74 715–729. 10.1111/tpj.12173 23489434

[B26] Jiang J. (2019). Fluorescence in situ hybridization in plants: recent developments and future applications. *Chromosome Res.* 27 153–165. 10.1007/s10577-019-09607-z 30852707

[B27] Karafiátová M. Hřibová E. Doležel J. (2017). “Cytogenetics of Cicer,” in *The Chickpea Genome*, eds Varshney R. Thudi M. Muehlbauer F. (Cham: Springer), 25–41. 10.1007/978-3-319-66117-9_4

[B28] Li H. Durbin R. (2010). Fast and accurate long-read alignment with burrows–wheeler transform. *Bioinformatics* 26 589–595. 10.1093/bioinformatics/btp698 20080505 PMC2828108

[B29] Liu X. Sun S. Wu Y. Zhou Y. Gu S. Yu H. (2020). Dual-color oligo-FISH can reveal chromosomal variations and evolution in *Oryza* species. *Plant J.* 101 112–121. 10.1111/tpj.14522 31494982

[B30] Ohri D. Pal M. (1991). The origin of chickpea (*Cicer arietinum* L.): karyotype and nuclear DNA amount. *Heredity* 66 367–372. 10.1038/hdy.1991.46

[B31] Parween S. Nawaz K. Roy R. Pole A. K. Suresh B. V. Misra G. (2015). An advanced draft genome assembly of a desi type chickpea (*Cicer arietinum* L.). *Sci. Rep.* 5:12806. 10.1038/srep12806 26259924 PMC4531285

[B32] Quinlan A. R. Hall I. M. (2010). BEDTools: a flexible suite of utilities for comparing genomic features. *Bioinformatics* 26 841–842. 10.1093/bioinformatics/btq033 20110278 PMC2832824

[B33] Rajesh P. N. Sant V. J. Gupta V. S. Muehlbauer F. J. Rajesh P. K. (2003). Genetic relationships among annual and perennial wild species of *Cicer* using inter simple sequence repeat (ISSR) polymorphism. *Euphytica* 129 15–23. 10.1023/A:1021567821141

[B34] Redden R. J. Berger J. D. (2007). “History and origin of chickpea,” in *Chickpea Breeding and Management*, eds Yadav S. S. Redden R. J. Chen W. Sharma B. (Oxfordshire: CAB International), 10.1079/9781845932138.001

[B35] Roorkiwal M. Jarquin D. Singh M. K. Gaur P. M. Bharadwaj C. Rathore A. (2018). Genomic-enabled prediction models using multi-environment trials to estimate the effect of genotype× environment interaction on prediction accuracy in chickpea. *Sci. Rep.* 8 1–11. 10.1038/s41598-018-30027-2 30076340 PMC6076323

[B36] Roorkiwal M. von Wettberg E. J. Upadhyaya H. D. Warschefsky E. Rathore A. Varshney R. K. (2014). Exploring germplasm diversity to understand the domestication process in *Cicer* spp. using SNP and DarT markers. *PLoS One* 9:e102016. 10.1371/journal.pone.0102016 25010059 PMC4092095

[B37] Ruperao P. Chan C. K. K. Azam S. Karafiátová M. Hayashi S. Čížková J. (2014). A chromosomal genomics approach to assess and validate the desi and kabuli draft chickpea genome assemblies. *Plant Biotechnol. J.* 12 778–786. 10.1111/pbi.12182 24702794

[B38] Schwarzacher T. Heslop-Harrison P. (2000). *Practical in Situ Hybridisation.* Oxford: BIOS Scientific Publishers Limited, 203.

[B39] Sharma P. C. Winter P. Bünger T. Hüttel B. Weising K. Kahl G. (1995). Abundance and polymorphism of di-, tri- and tetra-nucleotide tandem repeats in chickpea (*Cicer arietinum* L.). *Theor. Appl. Genet.* 90 90–96. 10.1007/BF00221000 24173788

[B40] Šimoníková D. Němečková A. Čížková J. Brown A. Swennen R. Doležel J. (2020). Chromosome painting in cultivated bananas and their wild relatives (*Musa* spp.) reveals differences in chromosome structure. *Int. J. Mol.* 21:7915. 10.3390/ijms21217915 33114462 PMC7672600

[B41] Šimoníková D. Němečková A. Karafiátová M. Uwimana B. Swennen R. Doležel J. (2019). Chromosome painting facilitates anchoring reference genome sequence to chromosomes in situ and integrated karyotyping in banana (*Musa* Spp.). *Front. Plant Sci.* 10:1503. 10.3389/fpls.2019.01503 31824534 PMC6879668

[B42] Staginnus C. Desel C. Schmidt T. Kahl G. (2010). Assembling a puzzle of dispersed retrotransposable sequences in the genome of chickpea (*Cicer arietinum* L.). *Genome* 53 1090–1102. 10.1139/G10-093 21164541

[B43] Staginnus C. Huettel B. Desel C. Schmidt T. Kahl G. (2001). A PCR-based assay to detect En/Spm-like transposon sequences in plants. *Chromosome Res.* 9 591–605. 10.1023/A:1012455520353 11721956

[B44] Staginnus C. Winter P. Desel C. Schmidt T. Kahl G. (1999). Molecular structure and chromosomal localization of major repetitive DNA families in the chickpea (*Cicer arietinum* L.) genome. *Plant Mol. Biol.* 39 1037–1050. 10.1023/A:1006125430386 10344208

[B45] Sudupak M. A. Akkaya M. S. Kence A. (2002). Analysis of genetic relationships omong perennial and annual *Cicer* species growing in Turkey using RAPD markers. *Theor. Appl. Genet.* 105 1220–1228. 10.1007/s00122-003-1505-8 12582902

[B46] Thudi M. Bohra A. Nayak S. N. Varghese N. Shah T. M. Penmetsa R. V. (2011). Novel SSR markers from BAC-end sequences, DArT arrays and a comprehensive genetic map with 1,291 marker loci for chickpea (*Cicer arietinum* L.). *PLoS One* 6:e27275. 10.1371/journal.pone.0027275 22102885 PMC3216927

[B47] Thudi M. Chitikineni A. Liu X. He W. Roorkiwal M. Yang W. (2016). Recent breeding programs enhanced genetic diversity in both desi and kabuli varieties of chickpea (*Cicer arietinum* L.). *Sci. Rep.* 6 1–10. 10.1038/srep38636 27982107 PMC5159902

[B48] Upadhyaya H. D. Dwicedi S. L. Baum M. Varshney R. K. Udupa S. M. Gowda C. L. (2008). Genetic structure, diversity, and allelic richness in composite collection and reference set in chickpea (*Cicer arietinum* L.). *BMC Plant Biol.* 8:106. 10.1186/1471-2229-8-106 18922189 PMC2583987

[B49] Varshney R. K. Song C. Saxena R. K. Azam S. Yu S. Sharpe A. G. (2013). Draft genome sequence of chickpea (*Cicer arietinum*) provides a resource for trait improvement. *Nat. Biotechnol.* 31 240–246. 10.1038/nbt.2491 23354103

[B50] Varshney R. K. Thudi M. Roorkiwal M. He W. Upadhyaya H. D. Yang W. (2019). Resequencing of 429 chickpea accessions from 45 countries provides insight into genome diversity, domestication and agronomic traits. *Nat. Genet.* 51 857–864. 10.1038/s41588-019-0401-3 31036963

[B51] Vláčilová K. Ohri D. Vrána J. Číhalíková J. Kubaláková M. Kahl G. (2002). Development of flow cytogenetics and physical genome mapping in chickpea (*Cicer arietinum* L.). *Chrom. Res.* 10 695–706. 10.1023/A:1021584914931 12575797

[B52] Wang J. Liu W. Zhu D. Hong P. Zhang S. Xiao S. (2020). Chromosome-scale genome assembly of sweet cherry (*Prunus avium* L.) cv. Tieton obtained using long-read and Hi-C seqeuncing. *Hortic. Res.* 7:122. 10.1038/s41438-020-00343-8 32821405 PMC7395734

[B53] Xin H. Zhang T. Han Y. Wu Y. Shi J. Xi M. (2018). Chromosome painting and comparative physical mapping of the sex chromosomes in *Populus tomentosa* and *Populus deltoides*. *Chromosoma* 127 313–321. 10.1007/s00412-018-0664-y 29520650

[B54] Zatloukalová P. Hřibová E. Kubaláková M. Suchánková P. Šimková H. Adoración C. (2011). Integration of genetic and physical maps of the chickpea (*Cicer arietinum* L.) genome using flow-sorted chromosomes. *Chromosome Res.* 19 729–739. 10.1007/s10577-011-9235-2 21947955

[B55] Zimin A. V. Marçais G. Puiu D. Roberts M. Salzberg S. L. Yorke J. A. (2013). The MaSuRCA genome assembler. *Bioinformatics* 29 2669–2677. 10.1093/bioinformatics/btt476 23990416 PMC3799473

