# Supplementary figures and images for: Karyotype Differentiation in Cultivated Chickpea Revealed by Oligopainting Fluorescence in situ Hybridization
Source: Front Plant Sci. 2022 Jan 25;12:791303. doi: 10.3389/fpls.2021.791303 (PMC8822127; doi:10.3389/fpls.2021.791303)

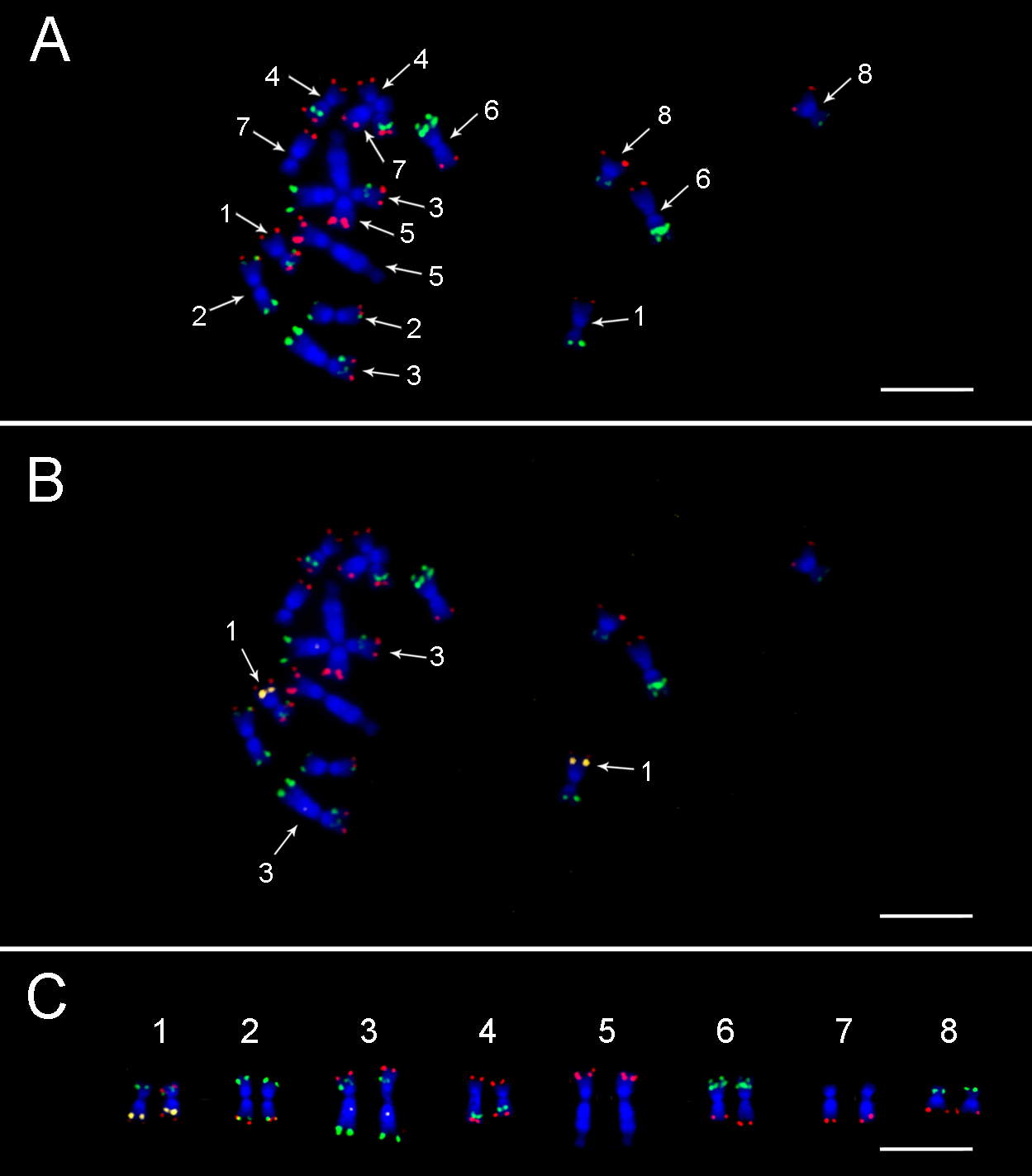

Supplement: Supplementary Figure 1 — ISH on mitotic metaphase plate of C. arietinum ICC 1882 (desi type) using (A) painting probes CAF-OP1 (green) and CAF-OP2 (red); (B) combination of the painting probes and 5S rDNA probe (yellow); and (C) molecular karyotype of ICC 1882 genotype (desi type). Chromosomes were counterstained with DAPI (blue). Bar = 3 μm. [file Image_1.TIF]

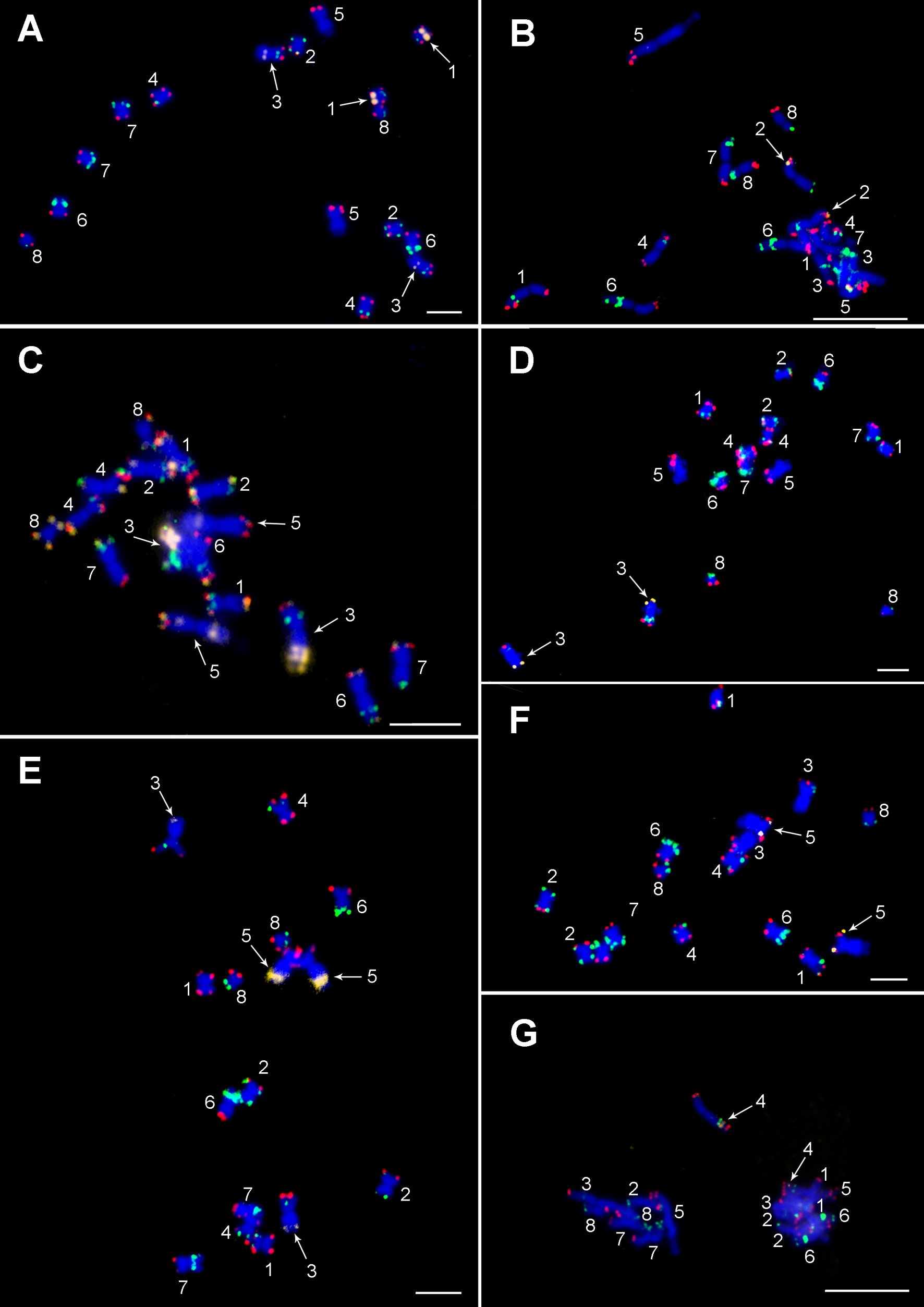

Supplement: Supplementary Figure 2 — Integration of oligopainting probe CAF-OP1 (green) and CAF-OP2 (red) with the extant cytogenetics landmarks (yellow) on mitotic metaphase chromosomes of C. arietinum CDC Frontier (kabuli): (A) 5S rDNA; (B) BAC 05E03; (C) telomeric sequence [TTTAGGG]n; (D) BAC 14M02; (E) 45S rDNA; (F) BAC 10I13; and (G) BAC 11K07, on mitotic metaphase chromosomes of C. arietinum CDC Frontier (kabuli). Chromosomes were counterstained with DAPI (blue). Arrows indicate positions of BAC clones or rRNA probes. Bar = 3 μm. [file Image_2.TIF]

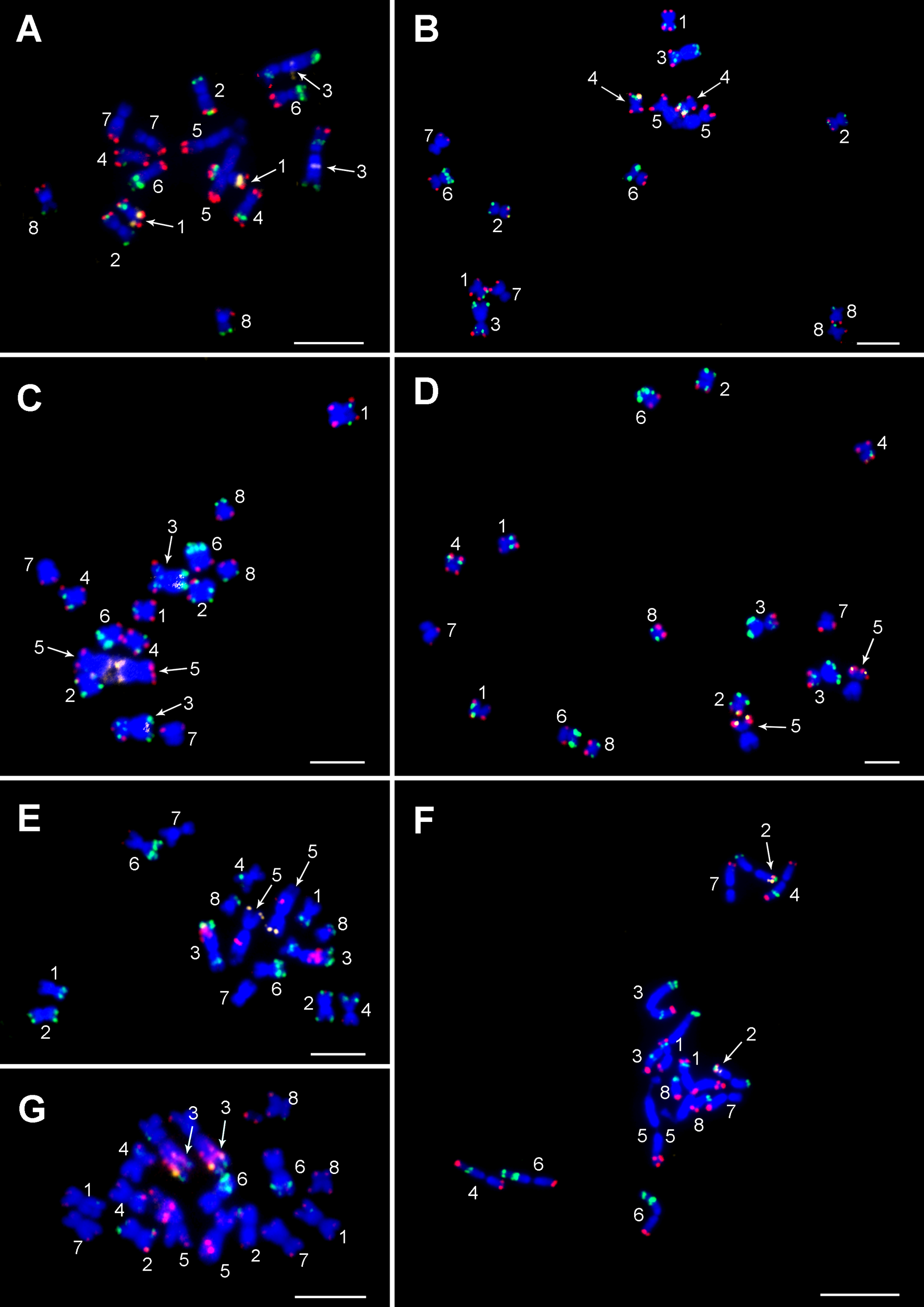

Supplement: Supplementary Figure 3 — Integration of oligopainting probe CAF-OP1 (green) and CAF-OP2 (red) with the extant cytogenetics landmarks (yellow) on mitotic metaphase plates of C. arietinum ICC 1882 (desi): (A) 5S rDNA; (B) BAC 11K07; (C) 45S rDNA; (D) BAC 10I13; and (F) BAC 5E03 (yellow). (E) Localization of oligopainting probe CAF-OP1 (green) with telomeric sequence (red) and BAC 10I13. (G) Colocalization of oligopainting probe CAF-OP1 (green) with telomeric sequence (red) and BAC 14M02 (yellow). Chromosomes were counterstained with DAPI (blue). Arrows indicate positions of BAC clones or rRNA probes. Bar = 3 μm. [file Image_3.TIF]
