# Supplementary material for: Karyotype Differentiation in Cultivated Chickpea Revealed by Oligopainting Fluorescence in situ Hybridization
Source: Front Plant Sci. 2022 Jan 25;12:791303. doi: 10.3389/fpls.2021.791303 (PMC8822127; doi:10.3389/fpls.2021.791303)
Supplement: Supplementary file 4 [file Table_1.DOCX]

**Supplementary Table 1.** Chromosome measurements of diploid *C. arietinum* CDC Frontier (kabuli type) and *C. arietinum* ICC 1882 (desi type).

| **Chromosome**  **(pseudomolecule)** | **Long arm (L) ± SE [µm]** | **Short arm (S) ± SE [µm]** | **Total lenght (L+S) ± SE [µm]** | **Arm ratio (L/S)** | **Chromosome morphology** |
| --- | --- | --- | --- | --- | --- |
| *Cicer arietinum* CDC Frontier (kabuli type) | | | | | |
| **1 (CaK1)** | 1.175 ± 0.148 | 0.895 ±0.163 | 2.070 | 1.313 | SM^a^ |
| **2 (CaK2)** | 1.165 ± 0.160 | 1.015 ±0.135 | 2.180 | 1.148 | M^b^ |
| **3 (CaK3)** | 1.810 ± 0.255 | 1.470 ±0.268 | 3.280 | 1.231 | SM |
| **4 (CaK4)** | 1.345 ± 0.119 | 0.980 ±0.185 | 2.330 | 1.372 | SM |
| **5 (CaK5)** | 2.330 ± 0.220 | 1.500 ±0.178 | 3.830 | 1.553 | SM + Sat^c^ |
| **6 (CaK6)** | 1.420 ± 0.151 | 1.145 ±0.139 | 2.570 | 1.240 | SM |
| **7 (CaK7)** | 1.265 ± 0.123 | 1.105 ±0.154 | 2.370 | 1.145 | M |
| **8 (CaK8)** | 0.950 ± 0.123 | 0.645 ±0.147 | 1.595 | 1.473 | SM |
| *Cicer arietinum* ICC 1882 (desi type) | | | | | |
| **1 (Ca1)** | 0.950 ± 0.239 | 0.870 ± 0.207 | 1.820 | 1.092 | M^a^ |
| **2 (Ca2)** | 1.020 ± 0.271 | 0.940 ± 0.250 | 2.210 | 1.085 | M |
| **3 (Ca3)** | 2.140 ± 0.470 | 1.440 ± 0.254 | 3.580 | 1.486 | SM^b^ |
| **4 (Ca4)** | 1.260 ± 0.350 | 0.950 ± 0.223 | 2.290 | 1.326 | SM |
| **5 (Ca5)** | 2.490 ± 0.542 | 1.480 ± 0.283 | 3.940 | 1.682 | SM+ Sat^c^ |
| **6 (Ca6)** | 1.320 ± 0.294 | 0.970 ± 0.249 | 2.290 | 1.361 | SM |
| **7 (Ca7)** | 1.320 ± 0.267 | 0.910 ± 0.150 | 2.230 | 1.451 | SM |
| **8 (Ca8)** | 0.770 ± 0.177 | 0.520 ± 0.128 | 1.290 | 1.481 | SM |

* Each chromosomal arm was measured in 10 metaphase cells.

^a^Sub-metacentric chromosome

^b^Metacentric chromosome

^c^Satellite chromosome
