# Supplementary material for: Karyotype Differentiation in Cultivated Chickpea Revealed by Oligopainting Fluorescence in situ Hybridization
Source: Front Plant Sci. 2022 Jan 25;12:791303. doi: 10.3389/fpls.2021.791303 (PMC8822127; doi:10.3389/fpls.2021.791303)
Supplement: Supplementary file 5 [file Table_2.PDF]

Table S2. Position of oligomer barcodes on the pseudomolecule of *C. arietinum* CDC Frontier, kabuli type.

Pseudomolecule	CAF-OP1, green painting probe			CAF-OP2, red painting probe		
	position [Mb]	oligos number	density per 1 kb	position [Mb]	oligos number	density per 1 kb
CaK1	43.60 – 44.11	1438	2.80	1.00 – 1.50	1864	3.70
				14.20 – 14.70	1789	3.60
CaK2	3.50 – 4.00	1718	3.40	32.70 – 33.20	1665	3.30
	35.80 -36.30	1656	3.30			
CaK3	16.80 – 17.30	1202	2.40	36.00 – 36.50	2108	4.20
CaK4	16.50 -17.00	1732	3.50	1.00 – 1.50	1933	3.90
				45.00 – 45.50	1654	3.30
CaK5				30.00 – 30.50	1731	3.50
				46.40 – 46.90	1765	3.50
CaK6	4.10 – 4.60	1754	3.50	57.60 – 58.11	1767	3.40
	19.00 – 19.50	1732	3.50			
	23.40 – 24.00	1996	3.30			
CaK7	2.70 – 3.20	2049	4.10	29.70 – 30.26	1700	3.00
	9.50 – 10.00	1744	2.00			
CaK8	14.40 – 17.90	1656	3.30	2.00 – 2.50	1941	3.90
		18677			19917	
